# Liver Enzymes and Inflammatory Markers Among Severely Ill COVID-19 Patients: A Retrospective Case-Control Study in Telangana

**DOI:** 10.7759/cureus.75120

**Published:** 2024-12-04

**Authors:** Pratyusha Pavuluri, M Girija Menon, Sarat Chandan Tummalacharla, Shaik Sameer Raheem, Soujanya Karpay, Phanindra Chepuri

**Affiliations:** 1 Biochemistry, RVM Institute of Medical Sciences and Research Center, Hyderabad, IND

**Keywords:** ace2 receptors, covid-19, cytokine storm, inflammatory markers, liver enzymes, systemic inflammation, viral load

## Abstract

Introduction

The COVID-19 pandemic originated in Wuhan, China, and swiftly spread across all continents. The respiratory system is the most affected in people who acquire sickness as a result of SARS-CoV-2. However, the virus can also affect other systems. The COVID-19 pandemic has become one of the most fatal infectious diseases in the recent past. Patients present with symptoms of fever, cough, tiredness, loss of taste or smell, sore throat, headache, and diarrhea.

Objective

This study intends to evaluate how COVID-19 has shown its effects on the well-being of the liver by collecting and correlating the data of the liver enzymes and inflammatory markers among hospitalized COVID-19 patients with age- and sex-matched healthy controls.

Materials and methods

A retrospective case-control study that included 200 patients diagnosed and hospitalized with COVID-19 was compared with an equal number of age- and sex-matched healthy control groups without COVID-19 at RVM Institute of Medical Sciences and Research Centre (RVMIMS & RC), a tertiary care teaching hospital in Siddipet, Telangana, India. Liver function tests (LFTs) and inflammatory markers were evaluated in both groups.

Results

Out of 200 patients, 179 (89.5%) had elevated alanine transaminase (ALT), 191 (95.5%) had elevated aspartate aminotransferase (AST), 33 (16.5%) had elevated alkaline phosphatase (ALP), and 183 (91.5%) showed elevated D-dimer levels. All the patients had elevated interleukin-6 (IL-6) and C-reactive protein (CRP) levels.

Conclusion

COVID-19 patients have exhibited elevations in liver enzyme panels and inflammatory markers. Further research and follow-up studies may aid in understanding the role of the well-being of the liver in patients affected by COVID-19. Considering the emergence of newer COVID-19 strains, we recommend LFT to patients who test positive for the virus to monitor prognosis and guide treatment protocols through this study.

## Introduction

The COVID-19 pandemic originated in Wuhan, China, and swiftly spread across all continents. The virus can affect multiple organ systems; however, the respiratory system is the most affected in individuals who develop severe illness as a result of SARS-CoV-2 infection [1]. COVID-19 is a highly contagious viral disease. The pandemic has become one of the most fatal infectious disease outbreaks in recent history. Patients typically present with symptoms such as fever, cough, fatigue, loss of taste or smell, sore throat, headache, diarrhea, and skin rashes [2,3]. The number of confirmed COVID-19 cases has exceeded 704 million worldwide, with fatalities surpassing seven million. In India, the total number of cases has surpassed 45 million, and the death toll exceeds 500,000 as of September 2024 [4].

The medical community has been working tirelessly to understand the damage caused by the coronavirus, as it affects multiple organ systems. Although the lungs are the most vulnerable, the virus also has a significant impact on the cardiovascular, gastrointestinal, renal, and central nervous systems [5]. COVID-19 is primarily transmitted via virus-laden droplets. The abundant presence of cell-surface receptors, such as angiotensin-converting enzyme 2 (ACE2) receptors in the lungs, makes these tissues particularly susceptible to infection. Once the virus enters the cell, it hijacks the cell's machinery to replicate itself and infect surrounding cells [6].

Liver function tests (LFTs) are among the most routinely performed diagnostic tests. These tests are used to investigate suspected liver diseases or monitor the progression of existing conditions, providing valuable information about a wide range of clinical phenomena. Alanine aminotransferase (ALT) and aspartate aminotransferase (AST) are enzymes that catalyze transamination reactions. ALT is found in higher concentrations in the liver, and any type of liver cell injury can lead to an increase in ALT levels. Elevated AST levels are seen in conditions such as myocardial infarction and liver tissue degeneration and necrosis. Alkaline phosphatase (ALP) is present in the small intestine, proximal convoluted tubule (PCT) of the kidney, bone, liver, and placenta. Mild elevation of ALP levels is observed in conditions like cirrhosis, hepatitis, and congestive heart failure [7].

Inflammatory mediators, such as cytokines and interleukins (IL)-6, perform both anti-inflammatory and pro-inflammatory activities. IL-6 plays a key role in the resolution of tissue damage, but its overproduction can lead to a cytokine storm. This excessive response has been linked to the severity and mortality of SARS-CoV-2-induced illness [8]. C-reactive protein (CRP) is a non-specific acute-phase reactant that is elevated during infection and inflammation. Increased CRP concentrations indicate a more intense inflammatory response [9]. D-dimer, a fibrin degradation product, is commonly used as a biomarker for thrombotic disorders. D-dimer levels increase with the severity of pneumonia. Following the outbreak of the COVID-19 pandemic, IL-6, CRP, and D-dimer have been used as potential indicators to assess the intensity and prognosis of COVID-19 illness [10].

The primary objective of this research is to understand the impact of SARS-CoV-2 on liver metabolism, with the goal of improving liver function and viability.

## Materials and methods

The present study is a retrospective analysis. Data were collected after obtaining approval from the Institutional Ethics Committee of RVM Institute of Medical Sciences and Research Centre (RVMIMS & RC) (IEC/RVMIMS&RC/2022/09/50). Information on hospitalized COVID-19 patients admitted during the months of April, May, and June 2021 was extracted from the Medical Records Department at RVMIMS & RC, a tertiary care teaching hospital located in Siddipet district, Telangana, India.

Method of data collection

Data were retrieved with the consent of hospital authorities. All patients included in the study were confirmed cases of COVID-19 by real-time reverse transcription-polymerase chain reaction (RT-PCR) and were age- and sex-matched with healthy controls.

Inclusion and exclusion criteria

Inclusion Criteria

All patients who provided verbal consent for participation, aged between 18 and 70 years, including both males and females, and diagnosed with COVID-19 were included in the study.

Exclusion Criteria

Patients with a documented history of obesity, chronic alcohol consumption, pregnancy or lactation, or comorbidities such as liver disease, diabetes mellitus, and immunodeficiency disorders were excluded from the study.

Study groups

Patients were divided into two groups: Group I (200 hospitalized COVID-19 patients) and Group II (200 healthy controls). Among the patient groups, 72% were men and 28% were women. All participants were tested for AST/SGOT, ALT/SGPT, ALP, IL-6, CRP, and D-dimer.

Laboratory tests

AST and ALT levels were estimated using the IFCC standard method, and ALP was estimated using the colorimetric method on the Randox Imola autoanalyzer.

The normal range of AST is up to 37 IU/L for males and up to 31 IU/L for females. The normal range of ALT is up to 40 IU/L for males and up to 31 IU/L for females. The normal range of ALP is 53-128 IU/L for males and 42-98 IU/L for females [11].

IL-6 levels were measured using a one-step immunoenzymatic "sandwich" method on the Beckman Access analyzer. The normal range for IL-6 is 5.3-7.5 pg/mL [12]. CRP levels were estimated using an immunoturbidometric assay on the Randox Imola autoanalyzer. The normal range for CRP is 0-5 mg/L [13]. D-Dimer levels were measured using fluorescence immunoassay technology on the Finecare POCT (point-of-care testing) instrument. The normal range for D-dimer is 0-500 ng/mL [14].

Statistical analysis

The retrieved data from the study participants were entered into Microsoft Excel 2024 (Microsoft Corporation, USA) for analysis. The data were presented as percentages, means, and standard deviations (SDs). A student’s t-test was performed to compare groups, and a p-value of <0.05 was considered statistically significant. IBM SPSS Statistics for Windows, version 22.0 (released 2013, IBM Corp., Armonk, NY) was used for data analysis.

## Results

A total of 200 patients were selected for the study. Among them, 144 (72%) were men between the ages of 22-70 years, and 56 (28%) were women between the ages of 21-70 years. The mean age of males was 54.6, and the mean age of females was 51.5. Demographic data are depicted in Table 1.

**Table 1:** Demographic data

Age group (years)	Male, 144 (72%)	Female, 56 (28%)
21-30	4 (2.8%)	6 (10.7%)
31-40	22 (15.3%)	7 (12.5%)
41-50	26 (18.1%)	14 (25.0%)
51-60	38 (26.4%)	17 (30.4%)
61-70	54 (37.5%)	12 (21.4%)
TOTAL	144	56

Out of the 200 patients, 179 (89.5%) had elevated ALT, 191 (95.5%) had elevated AST, 33 (16.5%) had elevated ALP, and 183 (91.5%) showed elevated D-dimer levels. All 200 patients exhibited elevated IL-6 and CRP levels.

The mean and SD of ALT in the patient group were 120.33 ± 67.47, while in the control group, it was 23.9 ± 6.4, with a significant p-value of ≤ 0.0001. The mean and SD of AST in the patient group were 124.93 ± 75.71, and in the control group, it was 13.8 ± 3.97, with a significant p-value of ≤0.0001. The mean and SD of ALP in the patient group was 96.62 ± 51.72, and in the control group, it was 82.01 ± 23.4, with a significant p-value of 0.0003. The mean and SD of IL-6 in the patient group were 534.03 ± 495.07, while in the control group, it was 6.5 ± 0.5, with a significant p-value of ≤0.0001. The mean and SD of CRP in the patient group were 107.37 ± 93.19, and in the control group, it was 2.5 ± 1.77, with a significant p-value of ≤0.0001. The mean and SD of D-dimer in the patient group were 824.1 ± 166.18, and in the control group, it was 244.07 ± 143.4, with a significant p-value of ≤0.0001. These findings indicate a positive correlation between COVID-19 illness and the elevation of liver enzymes and inflammatory markers. The results for the patient and control groups are summarized in Table 2.

**Table 2:** List of results for the liver enzymes and inflammatory markers in the patient and control groups ALT: alanine transaminase, AST: aspartate transaminase, ALP: alkaline phosphatase, IL-6: interleukin-6, CRP: C-reactive protein

Parameter	Cases (Group I)	Controls ( Group II)	P-value
	Mean ± SD	Mean ± SD	
ALT	120.33 ± 67.47	23.9 ± 6.4	≤0.0001
AST	124.93 ± 75.71	13.8 ± 3.97	≤0.0001
ALP	96.62 ± 51.72	82.01 ± 23.4	0.0003
IL-6	534.03 ± 495.07	6.5 ± 0.5	≤0.0001
CRP	107.37 ± 93.19	2.5 ± 1.77	≤0.0001
D-dimer	824.1 ± 166.18	244.07 ± 143.4	≤0.0001

## Discussion

The SARS-CoV-2 virus that caused COVID-19 was initially recognized as a pathogen responsible for pneumonia, later progressing to severe acute respiratory distress syndrome (ARDS). SARS-CoV-2-induced multi-organ dysfunction is associated with a range of hematologic abnormalities, cytokine storms, oxidative/nitrative stress, neurological symptoms, progressive renal failure, liver dysfunction, thrombosis, and septic shock [3]. This retrospective study focuses on the effects of COVID-19 on liver health.

Underlying causes of elevated liver enzymes and inflammatory markers

The primary causes for the elevation of liver enzymes and inflammatory markers in COVID-19 patients are 1) cytopathic impact via the ACE2 receptor, 2) immune-mediated inflammatory response (cytokine storm), and 3) septic shock.

Cytopathic Impact via the ACE2 Receptor

ACE2 receptors are expressed in various organs, including the liver, heart, kidneys, lungs, and central nervous system. The spike protein of SARS-CoV-2, a surface protein of the virus, binds to the ACE2 receptor, acting as a "gate" to enter human cells. Once inside, the virus hijacks the cellular machinery, replicates, destroys the host cell, and spreads to other cells. Although the lungs are a primary target for SARS-CoV-2 due to the abundance of ACE2 receptors, the liver also contains ACE2 receptors on hepatocytes, allowing the virus to infect liver cells and impair liver function [6].

Hypoxia, a common condition in COVID-19 patients, can increase ACE2 receptor expression in human hepatocytes. Given that many COVID-19 patients experience varying degrees of hypoxia and systemic inflammation, hepatocytes may become more susceptible to infection through the upregulation of ACE2 receptor expression under hypoxic conditions [15]. While evidence of ACE2 receptor expression in the liver has been found, the exact mechanism of liver injury in COVID-19 patients could result from direct viral infection or from a systemic inflammatory response syndrome (SIRS) [16].

In COVID-19 patients, elevated levels of angiotensin II have been observed, which correlate with viral load and the degree of lung injury. The complications of COVID-19 are exacerbated by the increased activity of the renin-angiotensin-aldosterone system (RAAS). In addition, antibody-dependent enhancement (ADE) of virus infection can occur, where virus-specific antibodies increase viral replication and enhance the ability of the virus to infect leukocytes by binding to Fc and complement receptors. ADE can mediate liver damage in an ACE2-independent manner, further contributing to liver dysfunction [17].

Immune-Mediated Inflammatory Response: Cytokine Storm and Septic Shock

A cytokine storm refers to the excessive activation of the immune system, which can be triggered by infection, certain drugs, or diseases. This results in the continuous activation and proliferation of lymphocytes and macrophages, leading to the rapid and massive secretion of inflammatory cytokines such as TNF-α, interferons (INF-α, INF-β, INF-γ), IL-1, IL-6, and IL-8. These cytokines cause severe organ damage through an inflammatory cascade [18]. In COVID-19 patients, this systemic inflammatory response can contribute to liver injury, acute lung injury, and ARDS by promoting a “cytokine storm” in severely ill patients.

As the immune system attempts to control and eradicate the virus, immunopathological damage occurs in tissues and organs. The release of excessive inflammatory cytokines leads to progressive tissue and organ damage. A cytokine storm is often associated with multiple organ dysfunction and can contribute to mortality [16,19].

IL-6, a pro-inflammatory cytokine, plays a central role in regulating the immune response. An exaggerated immune response, accompanied by increased IL-6 levels, is commonly observed in COVID-19 patients, leading to widespread inflammation and damage to organs, including the liver [20]. Although the exact mechanisms by which IL-6 contributes to liver injury are not fully understood, it is believed that the cytokine storm, systemic inflammation, and viral load directly affect hepatocytes. Monitoring IL-6, in conjunction with liver enzymes, can help assess liver involvement and predict patient prognosis [21-22].

CRP is another marker of systemic inflammation that can contribute to the cytokine storm in severe cases of COVID-19. Elevated CRP levels reflect the degree of the inflammatory response and can further impair liver function [23]. Liver injury in COVID-19 patients can thus result from various factors, including direct viral infection, cytokine storm, systemic inflammation, and drug-induced hepatotoxicity, which is reflected by elevated liver enzymes and inflammatory markers. This can lead to septic shock, the most common fatal complication in severe cases.

D-dimer and Liver Injury

D-dimer levels reflect a hypercoagulable state and fibrinolysis. Elevated D-dimer levels have been linked to liver injury in COVID-19 patients, although the exact mechanisms are still unclear. It is hypothesized that systemic inflammation and coagulation abnormalities contribute to liver dysfunction. In severe COVID-19 cases, elevated D-dimer levels are associated with an increased risk of thrombotic events, such as pulmonary embolism, disseminated intravascular coagulation (DIC), and microthrombi formation in the liver, further exacerbating liver injury [24-25].

Correlation with previous studies

In the current study, elevated IL-6 levels were strongly correlated with severe COVID-19 outcomes, which aligns with findings by Mojtabavi et al., who identified IL-6 as a useful biomarker for prognosis and disease severity [26]. Increased CRP levels observed in this study are consistent with the findings of Smilowitz NR et al., who showed that elevated CRP levels were strongly correlated with disease severity, ICU admission, and mortality, suggesting CRP as a potential predictive marker for clinical outcomes in COVID-19 patients [27]. Similarly, elevated D-dimer levels in our study are in agreement with those in the study of Phipps et al., who found that COVID-19 patients with elevated D-dimer levels exhibited severe liver injury and end-organ dysfunction. Their study also demonstrated a link between cytokine storm, inflammation, and liver damage [28].

Liver enzyme markers and disease severity

ALT is a specific marker of liver cell injury and can be important in assessing the severity of COVID-19. The present study found elevated ALT levels, which may be attributed to high viral load, systemic inflammation, and hepatocellular injury [15-16,29-30]. Elevated AST levels were also observed, correlating with the findings of Bhusal T et al. and Hayan Wu et al., who identified AST elevation as a reliable indicator of disease severity. This suggests that multi-organ damage caused by cytokine storms may contribute to abnormal AST levels. Although AST is not a specific marker of liver damage, its elevation indicates that immune-mediated inflammation plays a significant role in liver dysfunction in severe COVID-19 cases [15-16]. Notably, no significant elevation in ALP levels was observed in this study.

Limitations

This study has some limitations, including a relatively small sample size due to challenges encountered during data collection. Obtaining verbal consent was more difficult than anticipated, and the lack of a unique patient identification system in India posed a challenge for tracking liver metabolism abnormalities in COVID-19 patients. This limitation is particularly pronounced in rural areas, where the study was conducted.

## Conclusions

The present study demonstrates a threefold increase in AST and ALT levels in hospitalized SARS-CoV-2 patients, suggesting the presence of acute liver injury. In addition, the study shows elevated levels of inflammatory markers, such as IL-6, CRP, and D-dimer, in these patients, indicating a cytokine storm and systemic inflammation. Further research involving a larger patient cohort may provide a better understanding of the extent of liver damage and the potential long-term consequences on liver health due to SARS-CoV-2 infection. Given the continuous emergence of new SARS-CoV-2 strains, routine monitoring of these biomarkers can help healthcare providers assess disease severity, guide treatment protocols, and monitor patient recovery more effectively.
